# Engineering the Size of Bicontinuous Nanospheres via Multi-Inlet Vortex Mixing

**DOI:** 10.1021/acs.nanolett.5c04791

**Published:** 2025-12-04

**Authors:** Sultan Almunif, Simseok A. Yuk, El Hadji Arona Mbaye, Swagat Sharma, Michael D. Purdy, Sandeep Kumar, Natalie R. Klug, Evan A. Scott

**Affiliations:** † Department of Biomedical Engineering, 3270 Northwestern University, Evanston, Illinois 60208, United States; ‡ Department of Microbiology and Immunology, Feinberg School of Medicine, Northwestern University, Chicago, Illinois 60611, United States; § Department of Biomedical Engineering, NanoSTAR Institute, 12349 University of Virginia School of Medicine, Charlottesville, Virginia 22908, United States; ∥ Molecular Electron Microscopy Core, 12349 University of Virginia School of Medicine, Charlottesville, Virginia 22903, United States; ⊥ Bioengineering Institute, King Abdulaziz City for Science and Technology, Riyadh 11442, Saudi Arabia

**Keywords:** bicontinuous nanospheres, nanoparticles, self-assembly, flash nanoprecipitation, multi-inlet vortex mixer, protein corona

## Abstract

Bicontinuous nanospheres (BCNs) are self-assembled nanostructures with interconnected aqueous channels that enable the coloading of hydrophilic and hydrophobic cargo; however, their size has been difficult to control. Here, we present a scalable approach to tune the size distribution of poly­(ethylene glycol)-*b*-poly­(propylene sulfide) BCNs using a multi-inlet vortex mixer. Higher mixing times and polymer concentrations produced larger BCNs, while shorter mixing times and lower concentrations yielded spherical micelles. Small-angle X-ray scattering and cryogenic transmission electron microscopy confirmed the BCN bicontinuous morphology, which persisted at smaller sizes. The porous BCN structure resulted in increased surface roughness compared to polymersomes (PSs). *In vitro*, BCNs and PSs of comparable sizes recruited distinct protein coronas early, but their profiles showed convergence by 24 h. *In vivo*, organ biodistribution was determined primarily by the nanocarrier size rather than the morphology. These findings establish a robust approach to BCN fabrication while revealing dynamic biological interactions that inform nanocarrier design.

## Graphical abstract

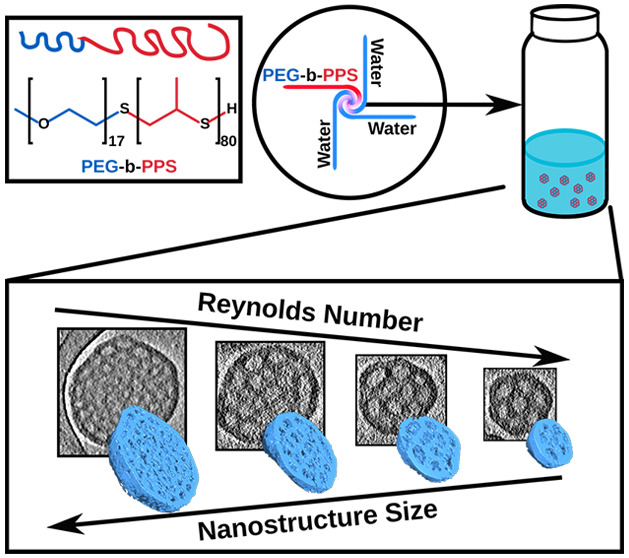

Nanocarriers are promising vehicles for the controlled delivery of vaccines and therapeutics.^1,2^ Considerable attention has been directed toward engineering nanocarriers for drug delivery applications using self-assembling amphiphilic block copolymers, where the hydrophobic/hydrophilic volume fraction plays a crucial role in determining the assembled nanostructures.^3–5^ The wide range of physiochemically distinct therapeutic molecules poses formulation challenges, especially when both hydrophilic and hydrophobic cargo must be coloaded without disrupting nanocarrier stability.^6–10^ Polymeric bicontinuous nanospheres (BCNs) are such nanostructures, but they remain largely untapped as a therapeutic nanocarrier platform due to difficulties in scalable fabrication while maintaining control over size distribution and internal structure.^11,12^ BCNs exhibit a distinct morphology consisting of a large hydrophobic volume that encloses a network of interconnected internal hydrophilic channels, enabling high loading of both hydrophobic and hydrophilic cargo.^11–13^ Depending on conditions, these internal networks may form highly ordered cubic phases or adopt more disordered sponge-like arrangements, but in all cases, they retain the bicontinuous feature of interconnected domains.^14–16^ We have demonstrated that the redox-responsive block copolymer poly­(ethylene glycol)-*b*-poly­(propylene sulfide) (PEG-*b*-PPS) can reproducibly assemble BCNs via flash nanoprecipitation (FNP).^5,13^ These nanostructures have subsequently been employed in a range of applications that leverage their unique morphology, stability, high drug loading efficiency, and dynamic properties.^12,13,19^ BCNs have been used to coencapsulate magnetic iron oxide nanostructures and molecular cargos while preserving their bicontinuous architecture, enabling enhanced magnetic resonance imaging contrast and oxidation-responsive morphological transitions.^17^ They also exhibit photo- and redox-triggered bicontinuous-to-micellar transitions that facilitate on-demand intracellular release of encapsulated therapeutics.^18^ Moreover, BCN formulations have enabled the delivery of both lipid and protein antigens for tuberculosis vaccination, achieving persistent lipid depots and robust antigen-specific T-cell responses.^19^ However, BCNs produced by this approach were 300–400 nm, limiting their utility for *in vivo* delivery.^12,13,19^ Additionally, methods such as thin film hydration failed to generate BCNs, while conventional solvent/antisolvent approaches produced less reproducible nanostructures and larger, more polydisperse BCNs.^13^

FNP is a scalable technique capable of producing uniformly spherical nanocarriers through rapid micromixing under supersaturated conditions, kinetically entrapping polymers into nanostructures.^20–23^ Among mixer designs, the multi-inlet vortex mixer (MIVM) offers greater control over solvent ratios and flow orientation than the confined impinging jet (CIJ), allowing higher supersaturation and more uniform particle formation.^20,24,25^ The size distributions of polymeric nanocarriers are significantly impacted by the mixing time and polymer aggregation balance.^20,21^ Controlling the size of the nanocarrier in a reproducible manner is essential in drug delivery and vaccine design, as it is one of the main indications of nanocarrier biodistribution.^26–28^ Generally, nanocarrier biodistribution is dependent on multiple variables that include surface chemistry, size, morphology, surface charge, and cell membrane/receptor interactions.^29–35^ With all variables being equal, the size still impacts *in vivo* biodistribution and cellular uptake, even when the morphology and surface chemistry are the same.^26–28^ The size-dependent biodistribution of nanocarriers across various mononuclear phagocytic system (MPS) organs highlights the importance of optimizing delivery platforms without relying on a standardized, one-size-fits-all solution.^36^ Therefore, specifying the size of nanocarriers that have a specific surface chemistry and morphology is critical for optimizing drug delivery. We have previously demonstrated that a modification in the chain length of PEG-*b*-PPS copolymers leads to various nanostructures that alter the organ- and cellular-level biodistribution.^12,32,33^ We have also demonstrated that these structures can be reproduced via FNP using CIJ while not affecting their ability to load drugs.^5^

PEG-*b*-PPS copolymer can form various nanostructures, including micelles, filomicelles, polymersomes (PSs), and BCNs, depending on the hydrophilic ratio (*f*_PEG_) and packing parameter (*p*).^5,12,13,37^ Because PEG-*b*-PPS BCNs incorporate a large PPS volume (∼75–80 repeating units), they adopt an inverted-phase morphology (*p* > 1), and low *f*_PEG_ values (∼0.11–0.12) favor such structures.^11,16^ Rapid stabilization of these long PPS chains is essential to prevent nanoparticle aggregation, a condition that FNP is well suited to achieve.^13,38^ Generating PEG-*b*-PPS BCNs using MIVM has not been reported; therefore, in this work, we investigated how manipulating mixing conditions with MIVM affects the BCN particle size, internal structure, and channel dimensions. We show that polymer concentrations and mixing times during MIVM can be used to tune the nucleation and growth of PEG_17_-*b*-PPS_80_ into BCNs of defined sizes. We employed an MIVM equipped with four inlets, allowing for greater variation in mixing. Unlike the CIJ used in our earlier work, the MIVM does not require equal volumes of solvent and antisolvent for effective mixing.^25,39,40^ One inlet was designated for the polymer dissolved in THF solution, while the other three inlets carried water or PBS as an antisolvent (Figure 1A,B). A 1:3 ratio of THF to water, compared to a 1:1 ratio in the CIJ mixer, increased the amount of the antisolvent, potentially enhancing the stability of the nanostructures by increasing the supersaturated concentration. By adjusting MIVM mixing velocities and polymer concentrations, we achieved control over nanostructure size and morphology without chemical modifications.

**Figure 1:**
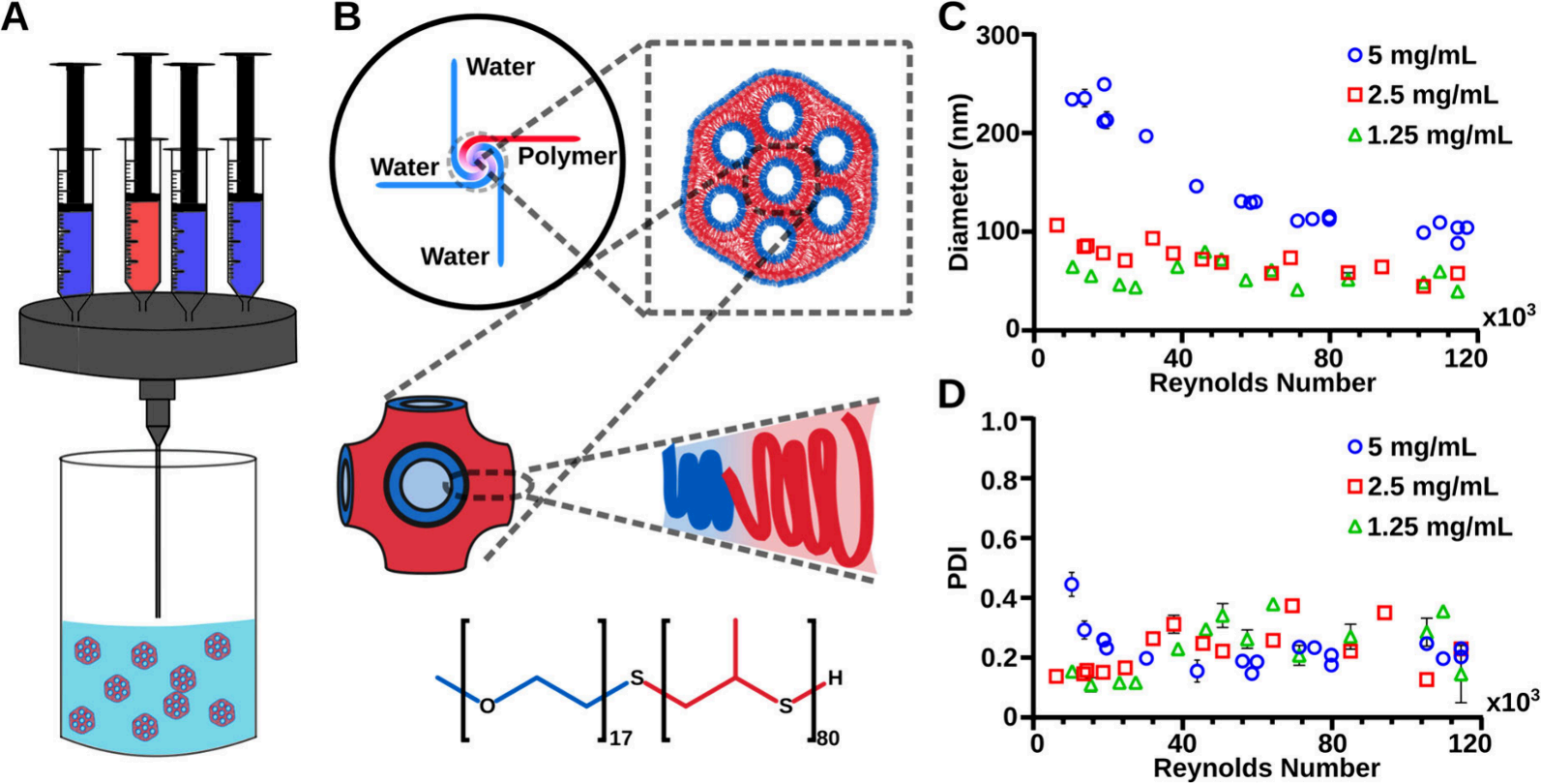
Self-assembly of bicontinuous nanospheres (BCNs) using a multi-inlet vortex mixer (MIVM). (A) Schematic of the MIVM design with one syringe inlet containing the polymer dissolved in a THF organic phase (red) and three containing the aqueous (water or PBS) antisolvent phase (blue). (B) Cross-sectional schematic of the MIVM with an illustration of the expected packing structure of the PEG_17_-*b*-PPS_80_ polymer within BCNs. (C) Hydrodynamic diameters of nanostructures measured by dynamic light scattering (DLS) with respect to the Reynolds number (*Re*). (D) Corresponding polydispersity index (PDI) values for each condition. Legends indicate the polymer concentrations used during the MIVM formulation.

Systematically decreasing the mixing velocity was found to increase the overall size of the nanostructures. This phenomenon can be attributed to the extended time available for the polymer to nucleate and assemble.^20,22,23^ Our experiments further demonstrated that the nanostructure size and morphology were concentration-dependent. We tested four different amounts of polymer (2.5, 5, 10, and 20 mg), which correspond to the following concentrations of 1.25, 2.5, 5, and 10 mg/mL, respectively, within the mixing chamber (Figure 1C, Figure S1A). For lower polymer concentrations, such as 1.25 and 2.5 mg/mL, the most dominant morphologies were solid core micelles with sizes ranging from 40 to 70 nm for the 1.25 mg/mL group and from 45 to 100 nm for the 2.5 mg/mL group, depending on the Reynolds number (*Re*) value (Figure 1C). The polydispersity index (PDI) for higher polymer concentrations, such as 5 and 10 mg/mL, decreased with increasing *Re* value (Figure 1D, Figure S1B). Lower polymer concentrations, such as 2.5 and 1.25 mg/mL, had PDI < 0.2 for low *Re* and fluctuating PDI for *Re* > 40,000. Although the size of the particles decreased with increasing *Re* at these lower concentrations, it was less sensitive to *Re* changes compared to higher polymer concentrations. Low polymer concentrations could not reach supersaturation states to aggregate into larger particles. Additionally, we found that solvent choice further modulated nanostructure size. PEG-*b*-PPS was only partially soluble in DMSO but more soluble in mixed DMSO/THF, with increasing DMSO content producing larger BCNs when the mixing time was held constant (Table S1, Figure S2). Together, these results demonstrate how polymer concentration, mixing dynamics, and solvent conditions collectively govern the self-assembly pathway and final morphology of PEG-*b*-PPS nanostructures.

To validate our observations, we employed small-angle X-ray scattering (SAXS) and cryogenic transmission electron microscopy (cryo-TEM), which confirmed that the aggregate morphology depended on both the polymer concentration and the mixing velocity. BCNs were identified by a disordered bicontinuous (sponge-like) structure, evidenced by a single broad SAXS peak at *q* ∼ 0.023 Å^–1^ corresponding to a characteristic length scale of ∼27 nm.^41,42^ At polymer concentrations below 5 mg/mL, the SAXS profiles fit well to a polymeric micellar model, a result supported by cryo-TEM (Figure 2A,B), with micelle sizes typically ranging from 40 to 70 nm (Table S1). As the polymer concentration increased and the *Re* value decreased, BCNs became increasingly prominent within the nanostructure population. Initially, a small fraction of BCNs were masked by the dominant micelle signal in SAXS, but at higher concentrations, a distinct SAXS peak confirmed a clear transition to a more complex internal structure (Figure 2A). The Porod region showed a power-law exponent between −3 and −4, indicating surface roughness consistent with the complex internal morphology of BCNs (Figure 2A).^43,44^ In contrast, at lower polymer concentration, the lower power-law exponent reflected the smoother surfaces of micelles compared to BCNs.^45^ Higher polymer concentrations and relatively longer mixing times favored a supersaturation state during FNP, prompting nucleation into a bicontinuous morphology. Conversely, higher impingement velocities reduced the effective mixing time, giving the polymer insufficient time to reorganize into a higher-order structure, thereby favoring micelle formation.

**Figure 2:**
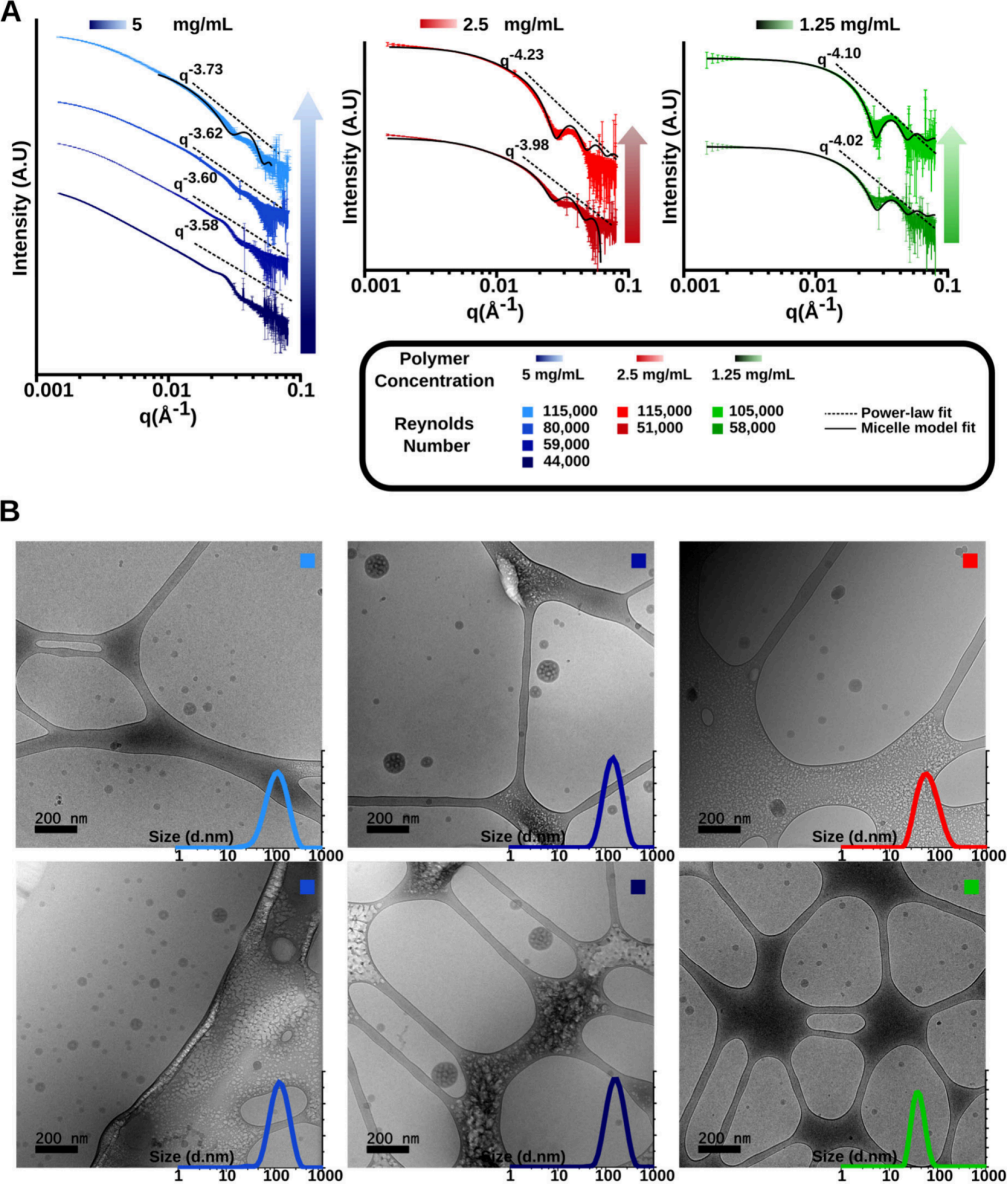
Characterization of PEG_17_-*b*-PPS_80_ nanostructures assembled via MIVM. (A) Small-angle X-ray scattering (SAXS) patterns demonstrating the formation of PEG_17_-*b*-PPS_80_ micelles and bicontinuous nanospheres (BCNs) as a function of the polymer concentration and Reynolds number (*Re*). The arrows indicate the direction of increasing *Re* value. Micelles were fitted to a polymeric micelle model by using SasView software. The power-law fit was applied in the Porod region of the SAXS profile. (B) Corresponding cryogenic transmission electron microscopy (cryo-TEM) images of the nanostructures for each concentration at various *Re* values. See the legend in (A) for sample details. The hydrodynamic diameters measured by dynamic light scattering (DLS) are overlaid on their respective cryo-TEM images.

To visualize the internal channels and assess whether they remain interconnected, we performed cryo-TEM tomography. A primary concern was that smaller BCNs might lose internal continuity; however, tomography confirmed that the connected channel network is preserved even at reduced particle sizes. Figure 3A shows four BCN size variants (100, 125, 170, and 240 nm) for which 3D reconstructions were obtained. Cross sections through the midplane of each rendered volume reveal continuous, interconnected pores (Figure 3B, Movies S1–S3). Both pore diameter (∼15–17 nm) and channel-to-channel spacing (∼25–28 nm, defined as the center-to-center distance between adjacent channels) remained consistent across all sizes, indicating that these structural features are independent of particle diameter. As a result, the total number of pores scaled proportionally with overall BCN size. This consistency reflects the fact that pore size is governed primarily by the polymer’s *f*_PEG_ ratio.^46^ Furthermore, encapsulation of a model protein (FITC–BSA) was unaffected by inlet configuration, with BCNs maintaining their internal nanostructure, consistent size (∼150 nm, PDI < 0.2), and ∼30% loading efficiency across all tested orientations (Figure S3). Together, these findings demonstrate that BCNs reliably maintain their internal morphology regardless of size, providing predictable internal volumes for coloading applications.

**Figure 3:**
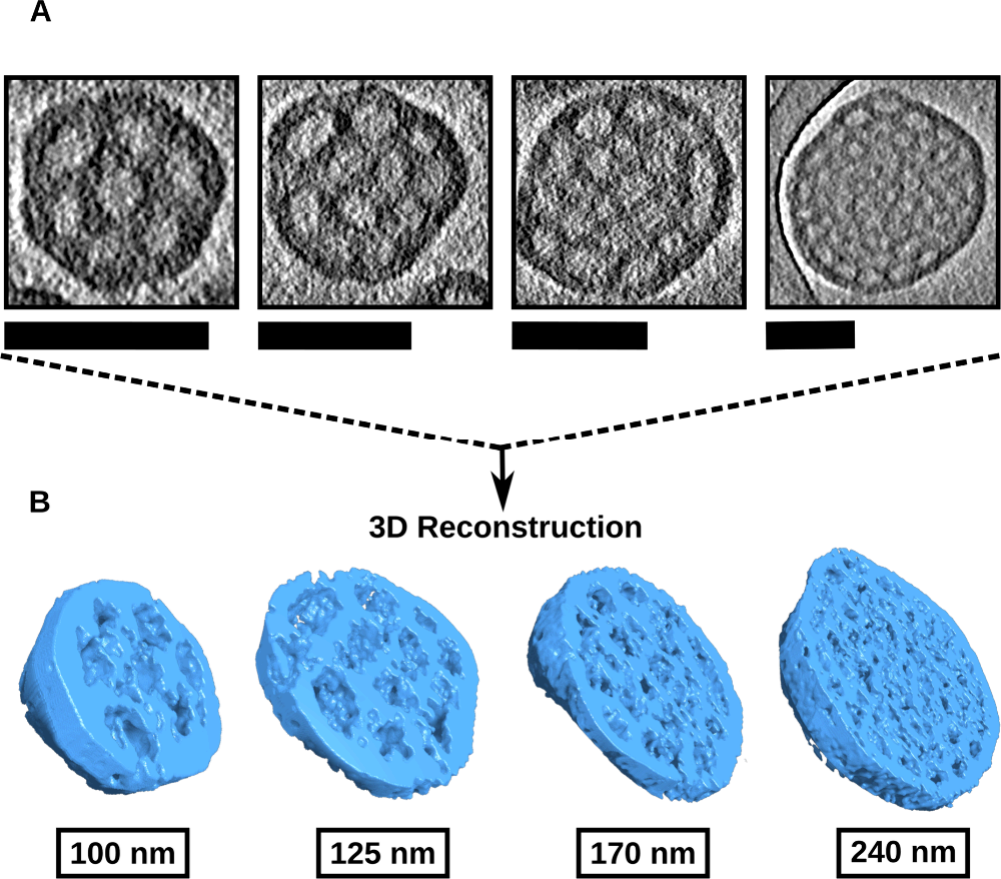
Tomographic analysis of BCNs of different sizes. (A) Cryo-TEM images of BCNs with varying diameters. Scale bar: 100 nm. (B) 3D reconstructions were generated from tomographic tilt series. Corresponding videos can be viewed in the Supporting Movies.

In Figure 2, SAXS results indicated that BCNs exhibit a power-law exponent greater than −4, consistent with surface irregularities, whereas micelles display slopes closer to −4, indicative of smoother interfaces. To investigate how these surface characteristics influence protein corona formation, we formulated an ∼150 nm PS using PEG_17_-*b*-PPS_35_ and a size-matched BCN (BCN150) using PEG_17_-*b*-PPS_80_ (Figure 4A,B, Table S2). We selected PSs rather than micelles as the smoother-surfaced control because the largest micelles we can stably prepare are <80 nm, whereas PSs allow stable monodisperse fabrication across a broader size range. Furthermore, both PS and BCN formulations share the same PEG block length, as confirmed by ^1^H NMR (Figure S4), ensuring a similar PEG density and surface chemistry. To evaluate the role of size independently of nanostructure morphologies, we also prepared larger BCNs (BCN200 and BCN300) (Figure 4B, Table S2). All nanocarriers were monodispersed (PDI ≤ 0.2) and exhibited comparable zeta potentials (Figure 4C, Table S2). Cryo-TEM confirmed their distinct morphologies (Figure 4D), and SAXS analysis supported bilayer formation in PS via vesicle model fitting (Figure 4E). Power-law exponents in the Porod region indicated smoother surface profiles for PS (slope ≈ – 4) compared to the rougher surfaces of BCNs (slope between −3 and −4; Figure 4E). To test the structural stability, we incubated nanocarriers in mouse plasma at 37 °C with agitation (220 rpm) for 2 and 24 h (Figure 4F). After 24 h, the BCN characteristic SAXS peak remained intact, indicating preserved internal structure (Figure 4G). In contrast, PS showed surface interference that impeded the vesicle model fitting. Additionally, we monitored the size and PDI of different BCNs over a 9 month period, which showed minimal changes, highlighting their long-term stability (Figure S5).

**Figure 4:**
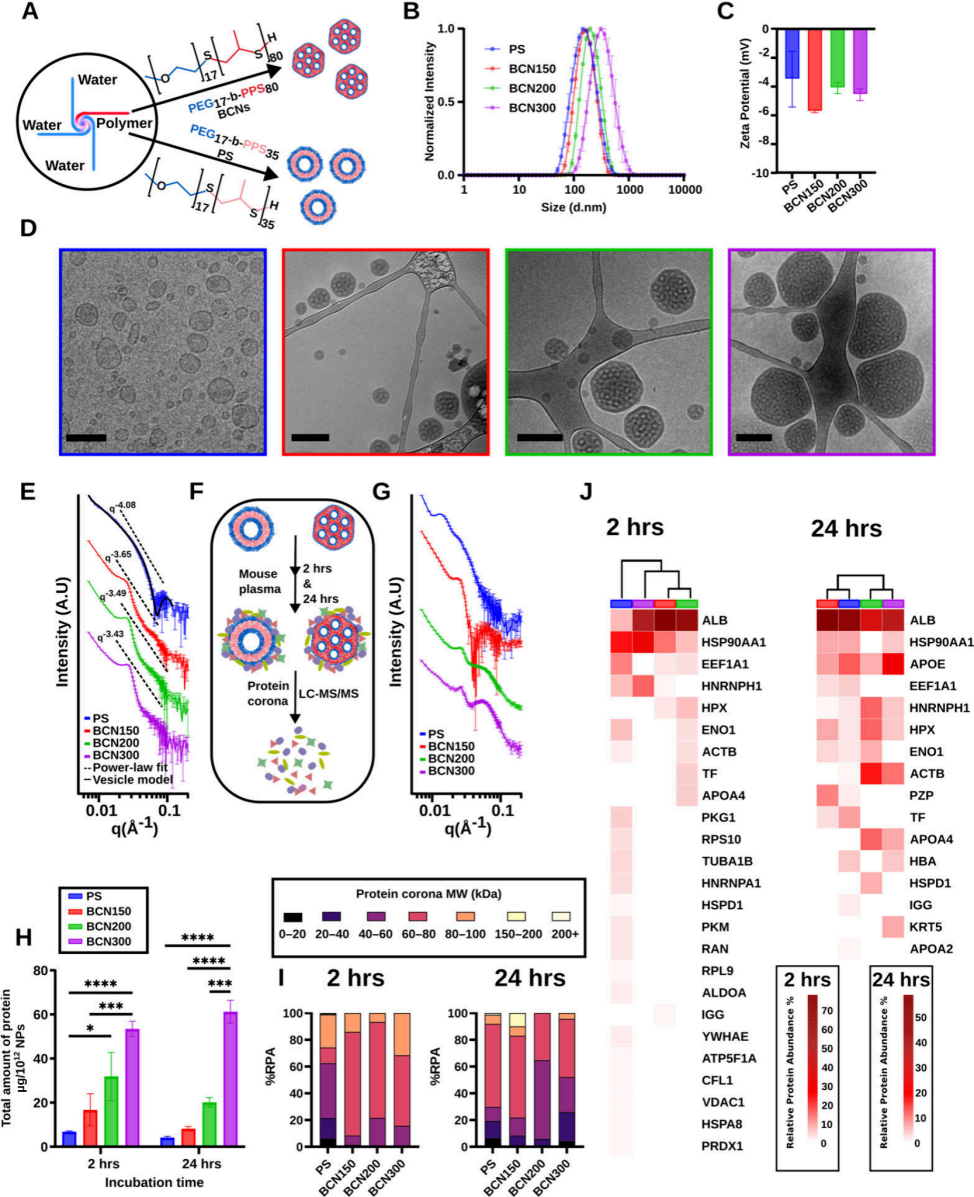
Physicochemical characterization and protein corona analysis of size-controlled BCNs and polymersomes (PSs). (A) Schematic illustration of MIVM generating BCNs using PEG_17_-*b*-PPS_80_ and PS using PEG_17_-*b*-PPS_35_. (B) Hydrodynamic diameters of PS and various BCN sizes measured by DLS. (C) Corresponding zeta potential. (D) Representative cryo-TEM images of each nanostructure (scale bar: 200 nm). (E) SAXS analysis of nanostructures. PS was fitted to a vesicle model using SasView software; a power-law fit was applied in the Porod region of each SAXS profile. (F) Experimental setup illustrating incubation of nanostructures with mouse plasma at two different time points followed by protein corona analysis. (G) SAXS profiles of nanostructures after 24 h of plasma incubation. (H) Total protein corona quantification was performed using the Pierce 660 nm Protein Assay. Statistical significance was measured by two-way ANOVA with post hoc Tukey’s test (*p < 0.05, **p < 0.01, ***p < 0.001, ****p < 0.0001). (I) Classification of protein corona composition by molecular weight (kDa) for each nanostructure at 2 and 24 h. (J) Hierarchical clustering of proteins constituting >1% of the total protein corona on nanostructure surfaces.

Following plasma incubation, unbound proteins were removed by multiple centrifugation steps, and total protein adsorption was quantified using the Pierce 660 nm Protein Assay. The data revealed a clear size-dependent trend, with BCN300 adsorbing significantly more protein than BCN200, BCN150, or PS at the two time points (Figure 4H). Label-free LC-MS/MS analysis revealed that early protein adsorption (2 h) on BCNs was dominated by proteins in the 60–80 kDa range, whereas PS primarily adsorbed proteins <60 kDa (Figure 4I). After 24 h, the protein distribution on PS shifted to resemble that of BCN150, suggesting a progressive protein exchange over time (Figure 4I). This temporal evolution was reflected in the hierarchical clustering of proteomic data: at 2 h, all BCNs clustered together, distinct from PS; by 24 h, PS and BCN150 grouped more closely, indicating a dynamic remodeling of the corona (Figure 4J). The heatmap also revealed key differences in corona composition. Albumin was the most abundant protein across all conditions and showed a strong binding to BCN surfaces. After 24 h, its relative abundance compared to the total protein corona composition decreased in all BCN coronas but increased on PS. This decrease on BCNs was accompanied by a greater adsorption of lipoproteins such as ApoE and ApoA, which are typically more stable corona constituents. This convergence is driven in part by the shift in PS toward larger adsorbed proteins and a relative albumin abundance comparable to that of BCN150, reflecting partial alignment in the protein size distribution and dominant corona constituents. Together, these findings suggest that nanostructure morphology influences early protein adsorption but that its influence diminishes over time, as PS and BCN150 protein corona compositions gradually converge, yielding a similar corona profile.

Nanocarriers varying in size and surface morphology (PS, BCN150, BCN200, and BCN300) were intravenously administered to C57BL/6 mice. Organs, including the spleen, liver, lung, kidneys, and heart, were harvested 24 h postinjection (Figure 5A). A major challenge in intravenous nanocarrier delivery is sequestration by MPS,^36,47^ primarily in the liver and spleen, where nanocarrier size plays a key role in biodistribution.^36,48^ Therefore, the ability to control size based on the intended application is critically important. Understanding how the nanocarrier size affects uptake by specific immune cell subsets is essential for optimizing drug delivery, enhancing targeting efficiency, and minimizing off-target clearance.

**Figure 5:**
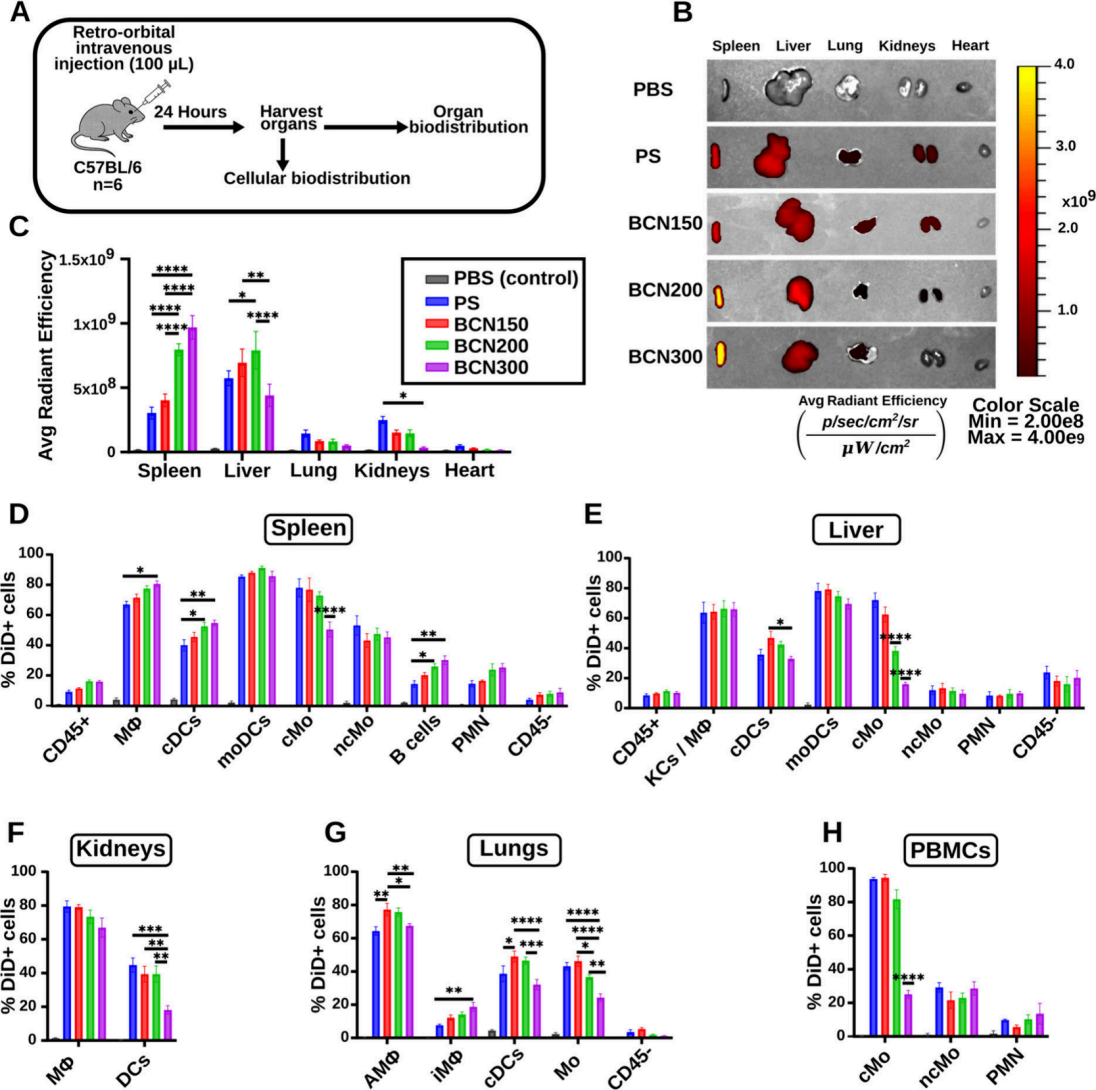
Nanocarrier biodistribution *in vivo* after intravenous injection. (A) Schematic illustration of retro-orbital intravenous injection (100 μL) of DiD-loaded nanocarriers (λem/λex = 640/680 nm) in C57BL/6 mice (*n* = 6 per group). (B) Representative *in vivo* imaging system (IVIS) images of organs collected 24 h postinjection. (C) Average radiance of DiD-loaded nanocarriers and control (PBS) in organs (spleen, liver, lung, kidneys, and heart). (D–H) Flow cytometric analysis of the cellular uptake of nanocarriers within the (D) spleen, (E) liver, (F) kidneys, (G) lungs, and (H) peripheral blood mononuclear cells (PBMCs). Abbreviations for cellular populations: leukocytes (CD45+), macrophages (MΦ), dendritic cells (DCs), conventional DCs (cDCs), monocyte-derived DCs (moDCs), classical monocytes (cMo), nonclassical monocytes (ncMo), polymorphonuclear neutrophils (PMN), Kupffer cells and macrophages (KCs/MΦ), alveolar macrophages (AMΦ), interstitial macrophages (iMΦ), nonleukocytes (CD45−). Each error bar represents SEM; *n* = 6 for tissues, *n* = 3 for PBMCs. Statistical significance was determined by two-way ANOVA with post hoc Tukey’s test (*p < 0.05, **p < 0.01, ***p < 0.001, ****p < 0.0001).

All nanocarriers were loaded with a hydrophobic dye (DiD) with encapsulation efficiencies ranging from 85% to 95% (Figure S6). Organ-level biodistribution was largely size-dependent and only minimally influenced by morphology. Larger nanocarriers (BCN200 and BCN300) accumulated significantly in the spleen, whereas the ∼150 nm PS and BCN150 showed similar, lower levels of splenic uptake. In the liver, the largest nanocarrier (BCN300) accumulated significantly less than the smaller nanocarriers (Figure 5B,C). This finding is consistent with our previous work, where BCN300 showed reduced liver uptake compared to PS (∼100 nm).^12^ Interestingly, BCN200 showed the highest liver accumulation, while PS and BCN150 had similar liver uptake (Figure 5C). PS also showed significantly greater kidney accumulation than BCN300. PS, BCN150, and BCN200 all exhibited greater accumulation in the lungs and kidneys compared to BCN300, suggesting that the nanocarrier size exerts a stronger influence over biodistribution than morphology. Overall, these results indicate that under the tested conditions, PS and BCN morphology do not substantially alter organ-level distribution within the same size range.

To explore cellular distribution across immune compartments, we prepared single-cell suspensions from the spleen, liver, kidneys, lung, and peripheral blood mononuclear cells (PBMCs) for flow cytometry (Figure 5D–H). Although the *in vivo* imaging system (IVIS) confirmed the uptake of nanocarriers, the flow cytometry data further support these differences at the cellular level. Gating strategies are listed in Table S3. In the spleen, size-dependent uptake was observed across antigen-presenting cells (APCs), including macrophages (MΦ), classical dendritic cells (cDCs), and B cells, with BCN300 showing significantly higher uptake than PS (Figure 5D). Conversely, classical monocytes (cMo) showed a significantly lower uptake of BCN300, with a clear preference for smaller nanocarriers. Interestingly, monocyte-derived dendritic cells (moDCs) showed high uptake across all nanocarrier types with no clear preference for size or morphology. In the liver, Kupffer cells, macrophages, and moDCs all showed high, nonspecific uptake of all nanocarriers (Figure 5E). As in the spleen, cMo cells exhibited size-specific uptake, favoring smaller carriers. cDCs in the liver also showed a higher uptake of BCN150 compared to BCN300. In comparing MΦ and DCs in the kidneys, DCs showed a significantly reduced uptake of BCN300, which may reflect the lower renal accumulation of BCN300 observed in IVIS imaging (Figure 5C,F). In the lung, PS showed the highest overall accumulation, as indicated by IVIS imaging, but BCN150 demonstrated significantly higher uptake in alveolar macrophages (AMΦ) and cDCs (Figure 5G). The lung was the only organ showing significant differences in uptake between the ∼150 nm PS and BCN150 morphologies. Consistent with observations in the spleen and liver, monocytes in the lung exhibited size-dependent uptake, with smaller nanocarriers being preferentially internalized. This pattern was also observed for PBMCs, where analysis of cMo, nonclassical monocytes (ncMo), and polymorphonuclear neutrophils (PMN) showed that only cMo displayed clear size-dependent uptake (Figure 5H). This may be explained by how nanocarriers are cleared by the immune system: larger nanocarriers are rapidly taken up by tissue-resident phagocytes (such as Kupffer cells in the liver and macrophages in the spleen),^48,49^ while smaller nanocarriers persist longer in circulation and are more likely to be internalized by circulating monocytes, especially during inflammation.^50,51^ Together, these results demonstrate that nanocarrier size strongly governs both organ-level biodistribution and immune cell uptake patterns *in vivo*, whereas nanocarrier morphology plays only a minor role under the tested conditions.

In this study, we fabricated BCNs of tunable sizes using MIVM, with *Re* as an indicator of the mixing efficiency. Higher *Re* values corresponded to shorter mixing times, which limited the available time for aggregate growth. Specifically, at a low polymer concentration, this favored the formation of simpler structures such as micelles rather than lyotropic BCNs. By contrast, increasing the polymer concentration while operating under lower *Re* promoted supersaturation, giving the polymer chain sufficient time to nucleate and grow into bicontinuous structures. By adjusting these parameters, we successfully controlled BCN size while confirming that even the smallest BCN retained its internal bicontinuous morphology. The enhanced control over BCN size achieved in this work establishes a unique and versatile nanocarrier platform with strong potential for site-specific drug delivery activated by modalities such as focused ultrasound.^17–19,52^ We further demonstrated that BCN morphology had a transient effect on early protein adsorption, with BCN showing distinct corona profiles compared to PS. *In vivo*, biodistribution was governed by size rather than morphology, underscoring that corona composition alone may not dictate organ-level fate.

## Supplementary Material





## Notes

Materials and experimental methods, summary of MIVM parameters and operating conditions, MIVM inlet orientation and protein loading, polymer characterization, BCN stability assessment, DiD encapsulation efficiency, turbidity analysis, and flow cytometry gating strategy (PDF)
Movies S1–S3: Cryo-TEM tomography videos (ZIP)
