# Supplementary material for: Engineering the Size of Bicontinuous Nanospheres via Multi-Inlet Vortex Mixing
Source: Nano Lett. 2025 Dec 4;25(50):17398–406. doi: 10.1021/acs.nanolett.5c04791 (PMC12715844; doi:10.1021/acs.nanolett.5c04791)
Supplement: Supplementary file 1 [file nl5c04791_si_001.pdf]

Engineering the Size of Bicontinuous Nanospheres via Multi-Inlet Vortex Mixing

Authors: Sultan Almunif[1,5], Simseok A. Yuk[1], El Hadji Arona Mbaye[1], Swagat Sharma[1], Michael D. Purdy[4], Sandeep Kumar[2], Natalie R. Klug[1], Evan A. Scott[1,3]*

Affiliations:

[1] Department of Biomedical Engineering, Northwestern University, Evanston, IL 60208, USA

[2] Department of Microbiology and Immunology, Feinberg School of Medicine, Northwestern University, Chicago, IL 60611, USA

[3] Department of Biomedical Engineering, NanoSTAR Institute, University of Virginia School of Medicine, Charlottesville, VA 22908, USA

[4] Molecular Electron Microscopy Core, University of Virginia School of Medicine, Charlottesville, VA 22903, USA

[5] Bioengineering Institute, King Abdulaziz City for Science and Technology, Riyadh, Saudi Arabia

[*] Corresponding author. Email: evan.scott@virginia.edu.

MATERIALS AND METHODS

Chemicals and reagents. Ethylenediaminetetraacetic acid disodium salt solution, Methanesulfonyl Chloride >=99.7%, Thiolacetic acid 96%, Dichloromethane, N,N-Dimethylformamide, triethylamine, Tetrahydrofuran, 0.5M Sodium Methoxide, Poly(ethylene glycol) methyl ether (average M_n 750), Heparin sodium salt, and Albumin from chicken egg white ≥98% were purchased from Sigma-Aldrich (St. Louis, MO, USA). Invitrogen™ eBioscience™ 1X RBC Lysis Buffer, Falcon™ Cell Strainers, DiD' solid; $DiIC_{18}(5)$ solid (1,1'-Dioctadecyl-3,3,3',3'-Tetramethylindodicarbocyanine, 4-Chlorobenzenesulfonate Salt), Falcon™ Round-Bottom Polystyrene Test Tubes with Cell Strainer Snap Cap 5mL, Propylene Sulfide (stabilized with Butyl Mercaptan) 98.0+%, Deepwell Mat 96 (1.2ml), Deepwell plate 96/500 µl, Heat-Inactivated Fetal Bovine Serum (FBS), Gibco Roswell Park Memorial Institute 1640 medium (RPMI), Gibco Dulbecco's Phosphate Buffered Saline (DPBS, without calcium chloride and magnesium chloride), BV480 anti-mouse CD3, BUV563 anti-mouse CD8α, and Pierce™ 660nm Protein Assay Reagent were purchased from Thermo Fisher Scientific (Waltham, MA, USA). Collagenase, Type 4 and Deoxyribonuclease I were purchased from Worthington Biochemical (Lakewood, New Jersey, USA). Mouse C57BL6 Plasma Na Citrate was purchased from Innovative Research Inc. Flow cytometry cell staining buffer (1x), anti-mouse CD16/CD32, fixable zombie near infrared viability dye and all remaining antibodies were purchased from BioLegend (San Diego, CA, USA).

Animals. All animal procedures were approved by Northwestern University's Institutional Animal Care and Use Committee, in compliance with the NIH guidelines for the care and use of laboratory animals. Female, 6-week-old C57BL/6 mice were purchased from the Jackson Laboratory (Bar Harbor, Maine, USA).

Synthesis and characterization of PEG-b-PPS. PEG_{17}-b-PPS_{80} and PEG_{17}-b-PPS_{35} were synthesized using methods previously described[1–3]. Initially, the hydroxyl group of polyethylene glycol methyl ether (mPEG) was converted to a mesylate by end-capping it with mesyl chloride. This step was followed by the substitution of the mesylate group with a thioacetate group through the functionalization of mPEG-mesylate using thioacetic acid. Ring-opening polymerization was then initiated by deprotonating mPEG-thioacetate with 0.5 M sodium methoxide, exposing the thiolate ion. Propylene sulfide was subsequently added and allowed to polymerize for 1 hour. The polymerization was terminated by adding acetic acid, which end-capped the polymer with a thiol group. The final polymer was purified by methanol precipitation followed by cold diethyl ether precipitation. The polymers were characterized by ^1H NMR ($CDCl_3$): δ= 1.30-1.40 (*m*, CH_3, PPS), 2.54-2.67 (*m*, CH, PPS), 2.71-2.75 (*t*, -CH_2S-, PEG), 2.81-2.97 (*m*, CH_2, PPS), 3.36-3.39 (*s*, OCH_3, PEG), 3.61-3.66 (*m*, CH_2, PEG); and by gel permeation chromatography (GPC) (Thermo Fisher Scientific). The retention times were 14.9 min for PEG_{17}-b-PPS_{80} and 16.5 min PEG_{17}-b-PPS_{35} retention time (**Figure S4**).

Nanostructure formulation and characterization. The self-assembly of PEG-b-PPS into nanostructures was achieved via a custom in-house constructed MIVM based on a published design[4]. Various amounts of PEG_{17}-b-PPS_{80} (2.5, 5, 10 or 20 mg) or PEG_{17}-b-PPS_{35} (20 mg) were dissolved in 500 µL of THF. The dissolved polymer solution was then loaded into a 1 mL Hamilton syringe and inserted into one of the four MIVM inlets. Each of the other three inlets were equipped with 1 mL Hamilton syringes filled with equal volumes of water (500 µL) (**Figure 1A**). In the mixing chamber, the organic and aqueous phases were impinged against each other, and the

resulting mixture was collected in a water reservoir (1 or 2 mL) in a 20 mL scintillation vial. The impinging velocity was regulated by a linear actuator connected to a plate that pressed down on the syringes, and the mixing time was recorded with a slow-motion camera. The mixing time is used to calculate the volumetric flow of the mixing process. The collected samples were then desiccated for 18-24 h to evaporate the organic solvent. For loading hydrophobic molecules, DiD dye was added to the same inlet containing the polymer in the organic phase. For loading hydrophilic molecules, FITC-BSA (100 µg) was dissolved in water and impinged against the polymer. The Reynolds number (Re) is calculated using the following expression:

$$Re = \frac{d}{A} \sum_{i}^{N} \frac{\rho_i Q_i}{\mu_i}$$

Where d is the chamber diameter (m), A is the inlet cross-sectional area (m^2), ρ is the fluid density (kg/m^3), Q is the volumetric flow (m^3/s), μ is the viscosity (Pa·s), and N is the total number of inlets.

The intensity distributions of the nanostructures' hydrodynamic diameter and the polydispersity index (PDI) were determined by Dynamic Light Scattering (DLS) using a Zetasizer Ultra Blue while the number distributions were determined via Nanoparticle Tracking Analysis (NTA) using NanoSight NS3000.

Small angle X-ray scattering (SAXS). Experiments were conducted at beamline 5-ID-D at the Dupont-Northwestern-Dow Collaborative Access Team Synchrotron Research Center (DND-CAT) at the Advanced Photon Source (APS) of Argonne National Laboratory, or at beamline 16-ID at the Life Science X-Ray Scattering (LIX) at the National Synchrotron Light Source II (NSLS-II) of Brookhaven National Laboratory. At beamline 5-ID-D, samples were analyzed in a 1.5 mm quartz capillary tube with a 10 keV collimated X-ray source (λ = 1.24 Å) with a sample to detector distance of 8.505 meters for the SAXS region: q-range of 0.0015 - 0.08085 $Å^{-1}$. At beamline 16-ID, samples were analyzed in a 2 mm thick well sealed between 100 um Ultra-thin Glass (G-Leaf™ Nippon) (https://github.com/stupplab/SAXScells). Data acquisition was performed using 15.5 keV collimated X-ray source for a continuous q-range of 0.005-3.2 $Å^{-1}$. The momentum transfer vector q is defined as $q = \frac{4\pi}{\lambda} \sin\theta$, where 2θ is the scattering angle. Data collected from 5-ID-D were processed using BioXTAS RAW[5] whereas data collected from 16-ID were processed using Lixtools (https://github.com/NSLS-II-LIX/lixtools). All data modeling was performed using SASview software. The power-law exponent was fitted using a python code written in house in a region where $q \gtrsim \frac{\pi}{Radius}$.

Cryogenic transmission electron microscopy (cryo-TEM). Before plunge-freezing, 200 mesh Cu grids with a lacey carbon membrane (EMS Cat# LC200-CU-100) were glow discharged using a Pelco easiGlow (Ted Pella) at 15 mA for 30 seconds under 0.24 mbar pressure, creating a negative charge on the carbon membrane to ensure even liquid sample distribution. 4 µL of sample (5 mg/mL) was applied to the glow discharged grid, blotted for 5 seconds with a blot offset of +1, and frozen by plunging into liquid ethane using FEI Vitrobot Mark IV. Grids were stored under liquid nitrogen. Grids were then loaded into a Gatan 626.6 cryo transfer holder, images were acquired at –175 °C in a JEOL JEM1400 LaB6 emission TEM at 120 kV, using a Gatan OneView 4k camera. Images were analyzed using ImageJ.

Cryo-TEM tomography. Lacey carbon or holey carbon (C-Flat 1.2/1.3) grids (Electron Microscopy Sciences) were glow-discharged for 45 s at 15 mA using a Pelco EasiGlow system. A Thermo Fisher Scientific Vitrobot Mark IV chamber was equilibrated to 4°C and 100% relative humidity, and a 3.5-µL sample aliquot was applied to each grid. Grids were blotted for 6-8 s with a blot force of 8 using Whatman No. 1 filter paper and vitrified by plunge-freezing in liquid ethane. Tilt-series data were acquired on a 200-keV Glacios transmission electron microscope equipped with a Falcon 4 direct electron detector (Thermo Fisher Scientific). Using TFS Tomography software (v. 5.14.0.5704REL), dose-symmetric tilt series were collected over a range of -60° to +60° in 3° increments at a target defocus of -4.0 µm. Data were recorded at a nominal magnification of 73,000x, corresponding to a physical pixel size of 1.9 Å (super-resolution pixel size, 0.95 Å). The dose per tilt image was 1.5 $e^-/Å^2$, for a total accumulated dose of 61 $e^-/Å^2$. Raw movie frames were processed using AreTomo3 (v.2.0.3)[6] to perform motion correction, generate aligned tilt series, and reconstruct tomograms. Motion correction was performed on 2x-binned frames in 5 x 5 patches, and the tilt series were aligned with 2x binning using full frames follow by local patch-based alignment (4 x 4 patches), resulting in a final pixel size of 3.8 Å. The alignment was calculated on a central 600-pixel-thick region. Tomograms with a final Z-height of 1,200 pixels were reconstructed by weighted back-projection. The reconstructed volumes were flipped along the y-axis to ensure consistent handedness. Reconstructed tomograms were visually inspected, and central slices were generated using the 3dmod viewer in the IMOD software package (v.4.11)[7]. The reconstructed tomograms were denoised using nonlinear anisotropic diffusion (NAD) filter in IMOD. The segmentation of the reconstructed tomograms, 3D volume visualization, and movie recording were carried out in Dragonfly (2024.1)[8].

Protein corona quantification and identification. Nanostructures were incubated 1:1 with mouse plasma for 2 h and 24 h at 37 °C, 220 RPM in Low Protein Binding Microcentrifuge Tubes (Thermo Fisher Scientific). After incubation, the samples were centrifuged at 15,000 × g for 40 minutes to remove unbound proteins. The pellets were washed twice with 1× PBS, each followed by centrifugation at 15,000 × g for 40 minutes. The total amount of adsorbed protein was quantified using the Pierce 660 nm protein assay, calibrated against bovine serum albumin (BSA). Subsequently, the adsorbed proteins were identified via label-free LC-MS/MS. Approximately 5 µg of protein was mixed with Laemmli buffer and 10% β-mercaptoethanol, then heated at 95 °C for 5 minutes prior to gel loading. The sample was subsequently loaded into a 4–20% Tris-glycine polyacrylamide gel. Electrophoresis was carried out at 120 V for ~5 minutes, achieving a migration distance of 3–5 mm from the well. A 1 cm × 1 cm gel region containing the protein mixture was excised. Proteins were in-gel digested with trypsin and desalted using ZipTip C18. The resulting peptide mixtures were analyzed by LC-MS/MS, with raw files searched against the *Mus musculus* (UniProt) database and common contaminants (e.g., trypsin, keratin, serum albumin) using FragPipe. For visualization, FragPipe results were imported into Scaffold DDA, and confident protein identification required at least two unique peptides per protein.

***In vivo* imaging system (IVIS).** For organ level biodistribution of BCNs, C57BL/6 female mice (6-week-old) were intravenously administered with either PBS, various sizes of DiD-loaded BCN, or DiD-loaded PS. The injection volume was 100 µL. The mice were euthanized 24 h post injections and blood (~500 µL) was collected retro-orbitally into heparinized tubes. Mice were then perfused with 5 mL heparinized PBS and tissues (liver, heart, kidneys, lung, and spleen) were harvested and imaged via IVIS Lumina with an excitation wavelength of 640 nm, an emission

wavelength of 680 nm, an exposure time of 0.25 s and an f/stop aperture of 2. *In vivo* images were analyzed using IVIS Lumina Living Image Software (v.4.5.5, PerkinElmer).

Flow cytometric analysis. Following IVIS imaging, liver, kidneys and lung were enzymatically digested with collagenase IV (1 mg/mL) and DNase I (30 µg/mL) at 37 °C, 30 minutes then mechanically dissociated through 70 µm filters using syringe plungers. Spleen samples were processed similarly but without enzymatic digestion. All samples were centrifuged (400 × g, 5 min) and the resulting pellets along with blood samples were then treated with red blood cell (RBC) lysis buffer for 5 minutes, stopped the lysis reaction with 1X PBS, and spun down (400 × g, 5 min). The single-cell suspensions were resuspended in Zombie Near Infrared for 15 minutes and subsequently washed twice. Anti-mouse CD16/CD32 was then added to the cells for FcR blocking for 15 minutes. Then, for 30 minutes, the cells were stained using cocktail of fluorophore-conjugated antibodies that consisted of PE-Fire 700 anti-mouse CD11c, BV421 anti-mouse CD11b, FITC anti-mouse Ly-6G, BV605 anti-mouse Ly-6C, BV650 anti-mouse MHC II (IA-IE), PE-Dazzle 594 anti-mouse F4/80, BV785 anti-mouse NK1.1, PerCP anti-mouse CD19, BUV563 anti-mouse CD8a, BV480 anti-mouse CD3, and BV570 anti-mouse CD45. The dilution of the above antibodies cocktail was done according to the manufacturer's recommendation. Finally, cells were washed twice with cell staining buffer and then resuspended in cell staining buffer. Data acquisition was performed using 3L 16V-14B-8R Aurora flow cytometer (CyTek). Both single color staining controls, and fluorescence minus one (FMO) controls were utilized to gate on the specific cellular populations. A minimum of 100,000 single, live cell events were recorded. SpectroFlo (CyTek) software was used for spectral unmixing, and FlowJo software was used for analysis.

Statistical analysis. All statistical analyses were performed using GraphPad Prism 10 (La Jolla, CA). Statistical significance was done by unpaired t-test or one/two-way ANOVA followed by Tukey's multiple comparisons test. A p-value of < 0.05 was considered statistically significant.

SUPPORTING FIGURES AND TABLES

Table S1. Summary of the parameters used to produce nanostructures using MIVM.

	Syringe depression time (sec)	PEG_{17}-b-PPS_{80} concentration (mg/mL)	Inlet 1 solvent	Inlet 2, 3 & 4	Inlet flow rate (mL/min)	Re	Diameter (nm)[a]	PDI[a]	Morphology[b]
**1**	2.52	1.25	THF	Water	12	10456	64.7	0.15	MC
**2**	0.68	1.25	THF	Water	44	38749	64.6	0.21	MC
**3**	0.46	1.25	THF	Water	65	57281	51.0	0.26	MC
**4**	0.31	1.25	THF	Water	97	84997	51.6	0.27	MC
**5**	0.25	1.25	THF	Water	120	105397	48.9	0.29	MC
**6**	0.23	1.25	THF	Water	130	114562	39.6	0.16	MC
**7**	4.27	2.5	THF	Water	7	6171	106.7	0.14	MC*, BCN
**8**	0.7	2.5	THF	Water	43	37642	78.0	0.31	MC*, BCN
**9**	0.52	2.5	THF	Water	58	50671	68.8	0.22	MC
**10**	0.31	2.5	THF	Water	97	84997	58.6	0.22	MC
**11**	0.23	2.5	THF	Water	130	114562	57.9	0.23	MC
**12**	0.25	2.5	THF	Water	120	105397	44.7	0.13	MC
**13**	1.92	5	THF	Water	16	18821	235.3	0.29	BCN
**14**	1.34	5	THF	Water	22	19664	213.2	0.23	BCN
**15**	0.6	5	THF	Water	50	43915	146.1	0.16	BCN
**16**	0.45	5	THF	Water	67	58554	129.4	0.15	MC, BCN
**17**	0.33	5	THF	Water	91	79846	114.9	0.18	MC, BCN
**18**	0.23	5	THF	Water	130	114562	104	0.20	MC*, BCN
**19**	1.83	10	THF	Water	16	14398	586.9	0.44	BCN, Agg
**20**	0.93	10	THF	Water	32	28332	275.5	0.2	BCN
**21**	0.63	10	THF	Water	48	50444	218.0	0.13	BCN
**22**	0.4	10	THF	Water	75	65873	179.6	0.07	BCN
**23**	0.35	10	THF	Water	86	75283	169.0	0.09	BCN
**24**	0.22	10	THF	Water	136	119769	139.5	0.11	BCN
**25**	0.24	5	DMSO:THF (1:4)	Water	125	94818	154.2	0.10	BCN
**26**	0.27	5	DMSO:THF (2:3)	Water	111	78393	193.7	0.10	BCN
**27**	0.25	5	DMSO:THF (3:2)	Water	120	81076	201.6	0.17	BCN
**28**	0.39	5	DMSO:THF(1:4)	Water	77	58350	164.7	0.11	BCN
**29**	0.48	5	DMSO:THF(1:4)	Water	63	47409	184.3	0.12	BCN
**30**	0.44	5	DMSO:THF(2:3)	Water	68	48105	286.7	0.23	BCN

MC (Micelles), BCN (Bicontinuous Nanospheres) Agg (Aggregates), * predominant morphology. [a] Determined by DLS. [b] Determined by Cryo-TEM and/or SAXS.

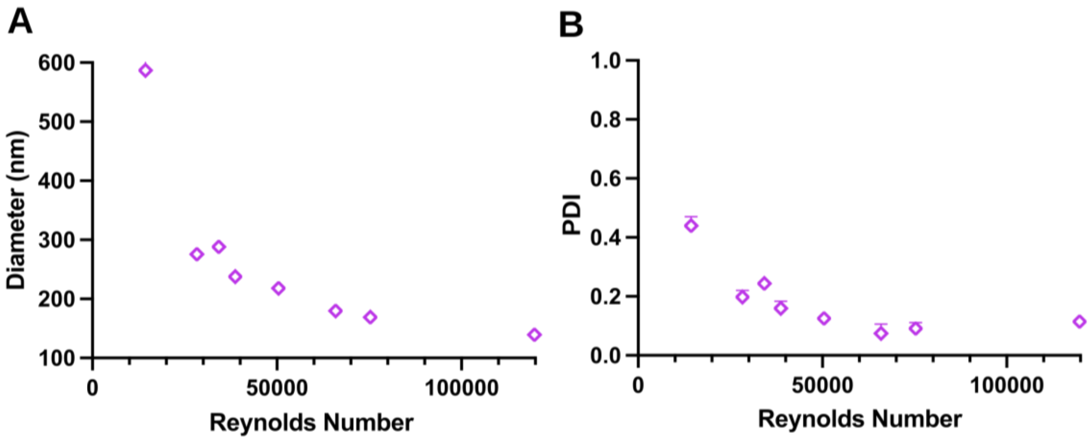

Figure S1. Bicontinuous nanospheres (BCNs) formation with 10 mg/mL of polymer using multi-inlet vortex mixer (MIVM). (A) hydrodynamic diameter and **(B)** polydispersity index (PDI) measured using dynamic light scattering (DLS).

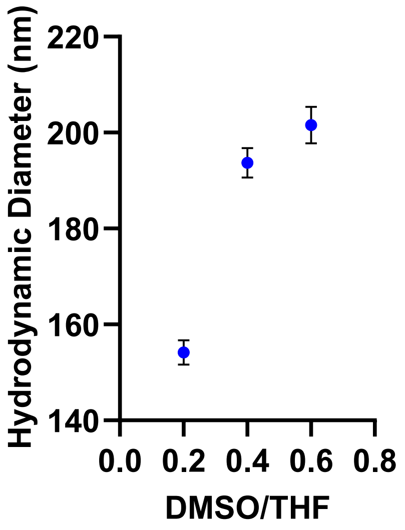

Figure S2. Bicontinuous nanospheres (BCNs) produced in a DMSO/THF mixture. The size of the BCNs increases as the DMSO increases at the same mixing velocity

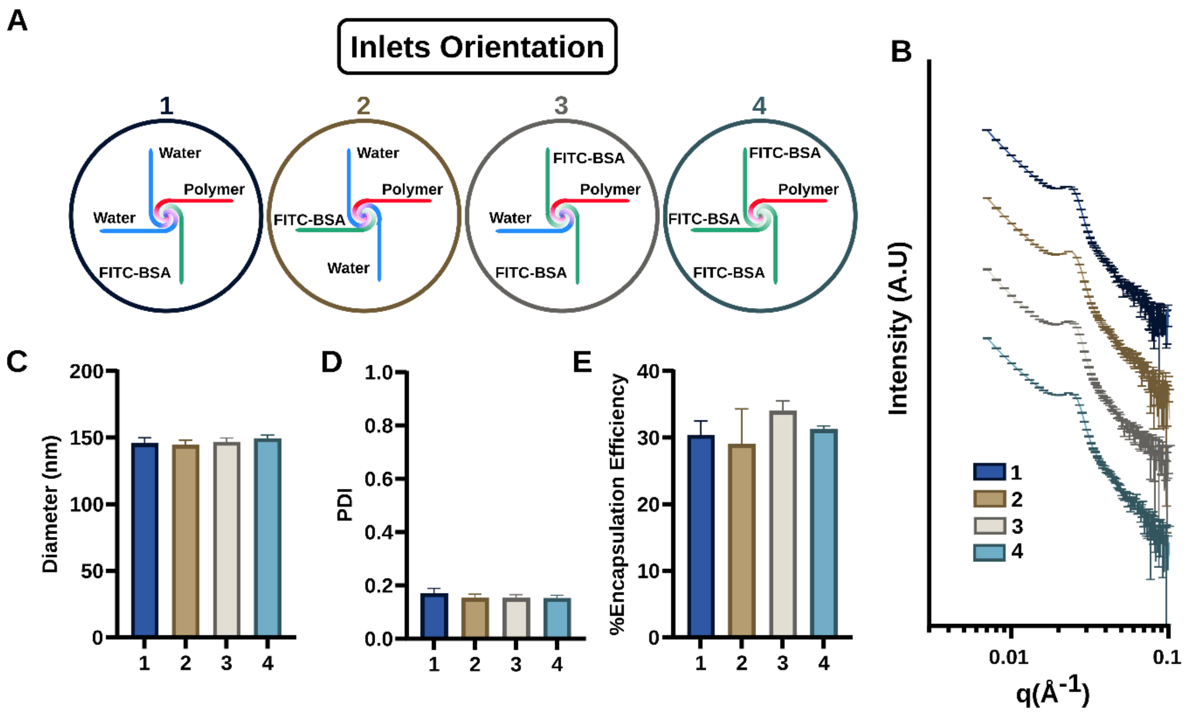

Figure S3. Encapsulation of FITC-labeled bovine serum albumin-FITC (FITC-BSA) within BCNs using different multi-inlet vortex mixing (MIVM) orientations. (A) Schematic illustrations of all MIVM inlet orientations tested. **(B)** SAXS profiles of BCNs generated under different MIVM orientations. **(C-D)** Hydrodynamic diameter and polydispersity index (PDI) of BCNs measured by DLS. **(E)** Encapsulation efficiency (%) of FITC-BSA in BCNs for each MIVM orientation. Error bars represent SEM.

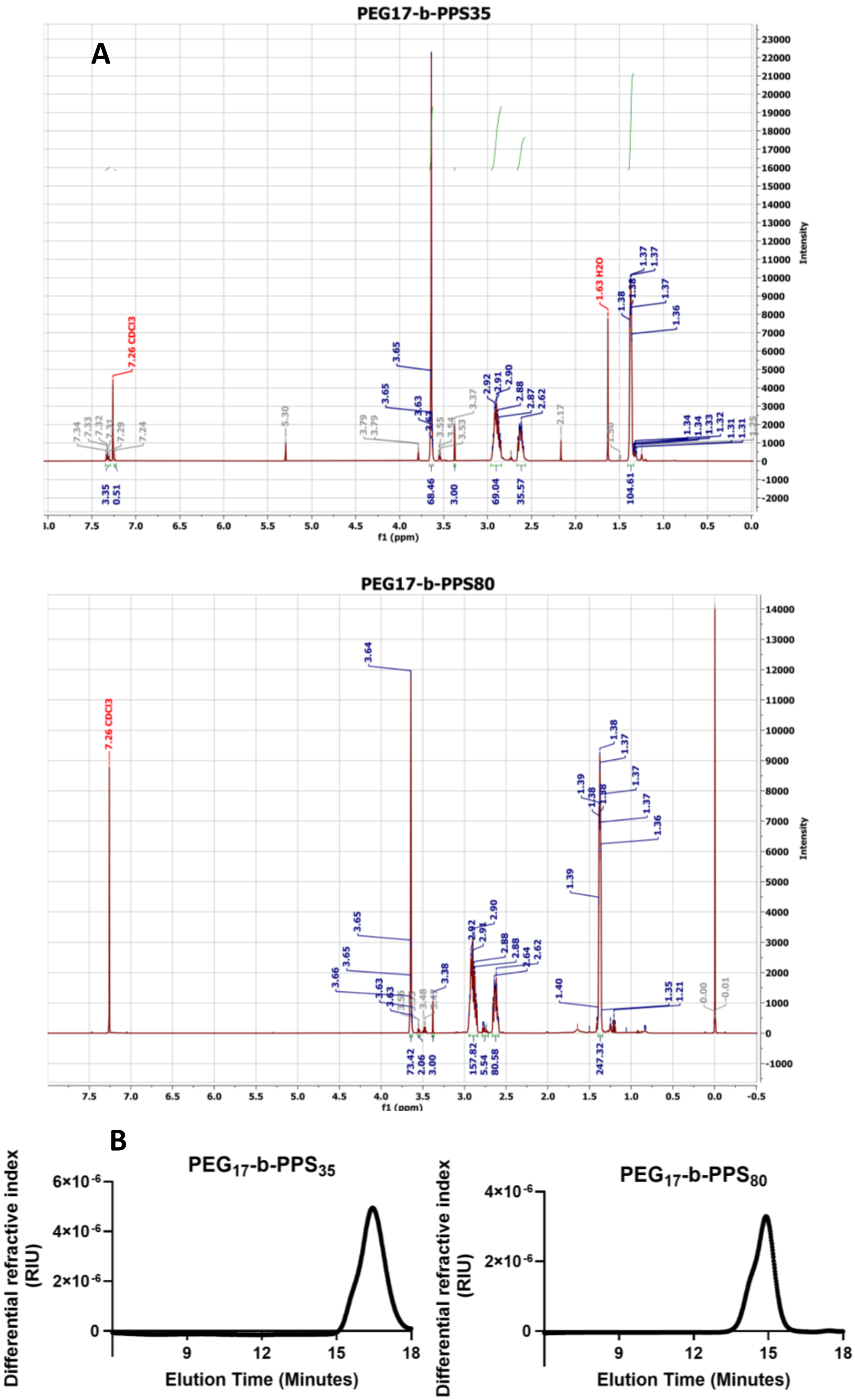

Figure S4. (A) ^{1}H NMR spectra of PEG$_{17}$-b-PPS$_{35}$ and PEG$_{17}$-b-PPS$_{80}$ copolymers and (**B**) their corresponding GPC profiles.

Table S2. Characteristics of PEG-b-PPS nanostructures formulated using MIVM.

	Polymer characterization					MIVM parameters				Nanostructure size	
Nanostructure	Polymer	Mn[a]	Mw[a]	Mn[b]	PDI[a]	Re ($\times 10^3$)	Polymer concentration (mg/mL)	Syringe depression time (sec)	Solvent	Diameter (nm)[c]	PDI[c]
PS	PEG_{17}-b-PPS_{35}	3598 ±49	4063± 50	3523	1.13± 0.02	61	10	0.43	THF	140.0±2.3	0.174
BCN150	PEG_{17}-b-PPS_{80}	6504 ±32	7151± 31	6832	1.10± 0.01	105	10	0.25	THF	151.7±4.1	0.114
BCN200						41	5	0.55	DMSO:THF (1:4)	195.1±3.6	0.121
BCN300						28	5	0.82	DMSO:THF (1:4)	306.8±4.8	0.216

[a] Determined by GPC. [b] Determined by ^{1}H NMR. [c] Determined by DLS.

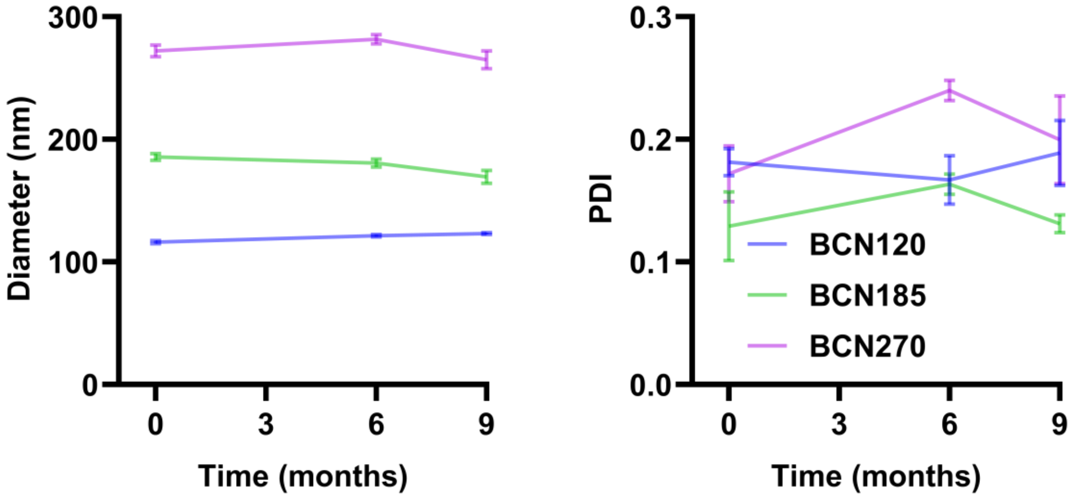

Figure S5. Various bicontinuous nanospheres (BCNs) size stability over nine months. (A) Hydrodynamic diameter, and **(B)** polydispersity index (PDI) measured by dynamic light scattering (DLS).

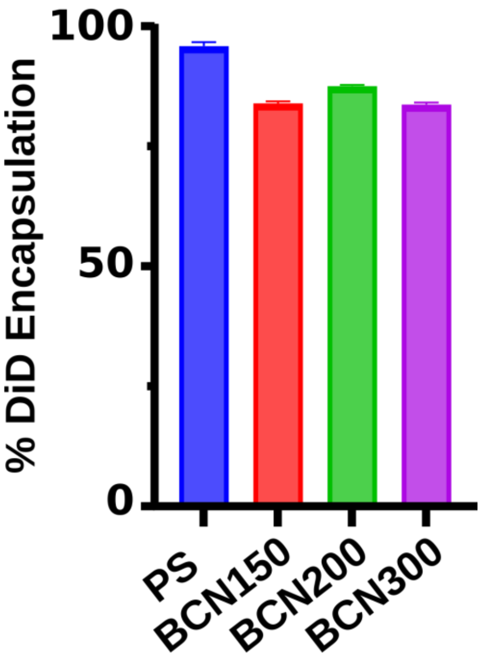

Figure S6. Nanocarriers encapsulation efficiency (%) of DiD dye.

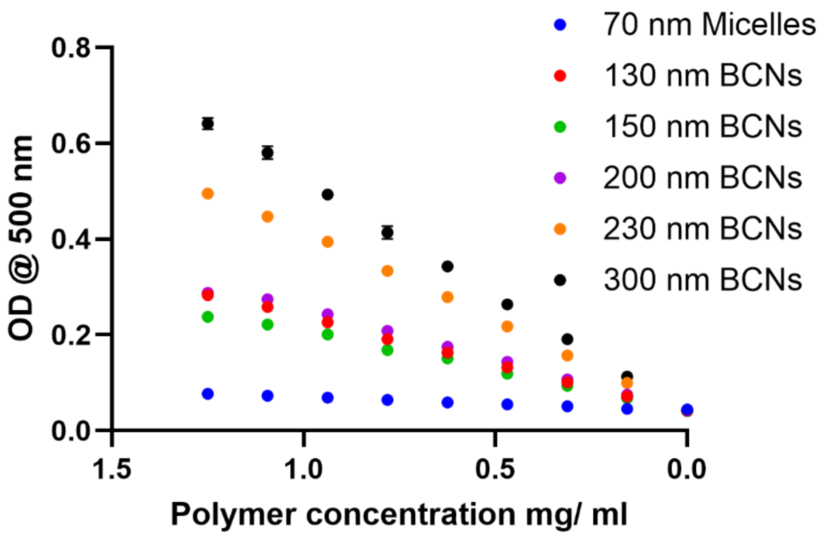

Figure S7. Turbidity analysis of nanostructures measured as optical density at 500 nm. The increase in turbidity is proportional to nanostructure size

Table S.3. Gating strategy of immune cells subpopulation within tissues.

**Peripheral Blood Mononuclear Cells (PBMCs)**	**Gating Strategy**
**Total Leukocytes**	$CD45^{+}$
**B Cells**	$CD45^{+} \rightarrow Ly6G^{-} \rightarrow CD19^{+}$
**T Cells**	$CD45^{+} \rightarrow Ly6G^{-} \rightarrow CD3^{+} \rightarrow NK1.1^{-}$
**Natural Killer T Cells**<br>**NK Cells**	$CD45^{+} \rightarrow Ly6G^{-} \rightarrow CD3^{-}\ CD19^{-} \rightarrow NK1.1^{+}$
**Classical Monocytes**<br>**(cMo)**	$CD45^{+} \rightarrow Ly6G^{-} \rightarrow CD3^{-}\ CD19^{-} \rightarrow NK1.1^{-} \rightarrow CD11b^{+} \rightarrow$ Ly6C^hi
**Non-Classical Monocytes**<br>**(ncMo)**	$CD45^{+} \rightarrow Ly6G^{-} \rightarrow CD3^{-}\ CD19^{-} \rightarrow NK1.1^{-} \rightarrow CD11b^{+} \rightarrow$ Ly6C^lo
**polymorphonuclear neutrophils**<br>**(PMN)**	$CD45^{+} \rightarrow Ly6G^{+} \rightarrow CD11b^{+}$
**Non-Leukocytes**	$CD45^{-}$
**Kidneys**	**Gating Strategy**
**Total Leukocytes**	$CD45^{+}$
**Macrophages (MΦ)**	$CD45^{+} \rightarrow Ly6G^{-} \rightarrow CD3^{-}\ CD19^{-} \rightarrow NK1.1^{-} \rightarrow F4/80^{+} \rightarrow CD11b^{+}$
**Dendritic Cells (DCs)**	$CD45^{+} \rightarrow Ly6G^{-} \rightarrow CD3^{-}\ CD19^{-} \rightarrow NK1.1^{-} \rightarrow F4/80^{-} \rightarrow CD11c^{+} \rightarrow MHCII^{+}$
**Non-Leukocytes**	$CD45^{-}$
**Lungs**	**Gating Strategy**
**Total Leukocytes**	$CD45^{+}$
**B Cells**	$CD45^{+} \rightarrow Ly6G^{-} \rightarrow CD19^{+}$
**T Cells**	$CD45^{+} \rightarrow Ly6G^{-} \rightarrow CD3^{+} \rightarrow NK1.1^{-}$
**Alveolar Macrophages (AMΦ)**	$CD45^{+} \rightarrow Ly6G^{-} \rightarrow CD3^{-}\ CD19^{-} \rightarrow NK1.1^{-} \rightarrow F4/80^{+} \rightarrow CD11c^{+} \rightarrow CD11b^{+} \rightarrow Ly6C^{-}$
**Interstitial Macrophages**<br>**(iMΦ)**	$CD45^{+} \rightarrow Ly6G^{-} \rightarrow CD3^{-}\ CD19^{-} \rightarrow NK1.1^{-} \rightarrow F4/80^{+} \rightarrow CD11c^{-} \rightarrow CD11b^{+} \rightarrow Ly6C^{-}$
**Dendritic Cells (DCs)**	$CD45^{+} \rightarrow Ly6G^{-} \rightarrow CD3^{-}\ CD19^{-} \rightarrow NK1.1^{-} \rightarrow F4/80^{-} \rightarrow CD11c^{+} \rightarrow MHCII^{+}$
**Monocytes**<br>**(Mo)**	$CD45^{+} \rightarrow Ly6G^{-} \rightarrow CD3^{-}\ CD19^{-} \rightarrow NK1.1^{-} \rightarrow F4/80^{-} \rightarrow CD11c^{-} \rightarrow CD11b^{+} \rightarrow Ly6C^{+}$
**polymorphonuclear neutrophils**<br>**(PMN)**	$CD45^{+} \rightarrow Ly6G^{+} \rightarrow CD11b^{+}$
**Non-Leukocytes**	$CD45^{-}$
**Liver**	**Gating Strategy**
**Total Leukocytes**	$CD45^{+}$
**B Cells**	$CD45^{+} \rightarrow Ly6G^{-} \rightarrow CD19^{+}$
**T Cells**	$CD45^{+} \rightarrow Ly6G^{-} \rightarrow CD3^{+} \rightarrow NK1.1^{-}$
**Natural Killer Cells**<br>**(NK Cells)**	$CD45^{+} \rightarrow Ly6G^{-} \rightarrow CD3^{-}\ CD19^{-} \rightarrow NK1.1^{+}$
**Dendritic Cells (DCs)**	$CD45^{+} \rightarrow Ly6G^{-} \rightarrow CD3^{-}\ CD19^{-} \rightarrow NK1.1^{-} \rightarrow F4/80^{-} \rightarrow CD11c^{+} \rightarrow MHCII^{+}$
**Monocyte-derived DCs**<br>**(moDCs)**	$CD45^{+} \rightarrow Ly6G^{-} \rightarrow CD3^{-}\ CD19^{-} \rightarrow NK1.1^{-} \rightarrow F4/80^{-/lo} \rightarrow CD11b^{+} \rightarrow CD11c^{+} \rightarrow Ly6C^{+} \rightarrow MHCII^{+}$
**Non-Classical Monocytes**<br>**(ncMo)**	$CD45^{+} \rightarrow Ly6G^{-} \rightarrow CD3^{-}\ CD19^{-} \rightarrow NK1.1^{-} \rightarrow F4/80^{-/lo} \rightarrow CD11b^{+} \rightarrow Ly6C^{lo}$
**Classical Monocytes**<br>**(cMo)**	$CD45^{+} \rightarrow Ly6G^{-} \rightarrow CD3^{-}\ CD19^{-} \rightarrow NK1.1^{-} \rightarrow F4/80^{-/lo} \rightarrow CD11b^{+} \rightarrow Ly6C^{hi}$
**Kupffer Cells and Macrophages (KCs/ MΦ)**	$CD45^{+} \rightarrow Ly6G^{-} \rightarrow CD3^{-}\ CD19^{-} \rightarrow NK1.1^{-} \rightarrow F4/80^{hi} \rightarrow CD11b^{lo} \rightarrow Ly6C^{-}$
**polymorphonuclear neutrophils**<br>**(PMN)**	$CD45^{+} \rightarrow Ly6G^{+} \rightarrow CD11b^{+}$
**Non-Leukocytes**	$CD45^{-}$

Spleen	Gating Strategy
**Total Leukocytes**	$CD45^+$
**B Cells**	$CD45^+ \rightarrow Ly6G^- \rightarrow CD19^+$
**T Cells**	$CD45^+ \rightarrow Ly6G^- \rightarrow CD3^+ \rightarrow NK1.1^-$
**Natural Killer T Cells (NKT Cells)**	$CD45^+ \rightarrow Ly6G^- \rightarrow CD3^+ \rightarrow NK1.1^+$
**Natural Killer T Cells (NK Cells)**	$CD45^+ \rightarrow Ly6G^- \rightarrow CD3^- \ CD19^- \rightarrow NK1.1^+$
**Monocyte-Derived Dendritic Cells (moDCs)**	$CD45^+ \rightarrow Ly6G^- \rightarrow CD3^- \ CD19^- \rightarrow NK1.1^- \rightarrow CD11b^+ \rightarrow CD11c^+ \rightarrow MHCII^+ \rightarrow Ly6C^+ \rightarrow F4/80^+$
**Classical Monocytes (cMo)**	$CD45^+ \rightarrow Ly6G^- \rightarrow CD3^- \ CD19^- \rightarrow NK1.1^- \rightarrow CD11b^+ \rightarrow Ly6C^{hi} \rightarrow F4/80^-$
**Non-Classical Monocytes (ncMo)**	$CD45^+ \rightarrow Ly6G^- \rightarrow CD3^- \ CD19^- \rightarrow NK1.1^- \rightarrow CD11b^+ \rightarrow Ly6C^{lo} \rightarrow F4/80^-$
**Macrophages (MΦ)**	$CD45^+ \rightarrow Ly6G^- \rightarrow CD3^- \ CD19^- \rightarrow NK1.1^- \rightarrow CD11b^+ \rightarrow Ly6C^- \rightarrow F4/80^+$
**Conventional DCs (cDCs)**	$CD45^+ \rightarrow Ly6G^- \rightarrow CD3^- \ CD19^- \rightarrow NK1.1^- \rightarrow CD11c^+ \rightarrow MHCII^+ \rightarrow F4/80^-$
**polymorphonuclear neutrophils (PMN)**	$CD45^+ \rightarrow Ly6G^+ \rightarrow CD11b^+$
**Non-Leukocytes**	$CD45^-$

References:

(1) Allen, S. D.; Bobbala, S.; Karabin, N. B.; Modak, M.; Scott, E. A. Benchmarking Bicontinuous Nanospheres against Polymersomes for in Vivo Biodistribution and Dual Intracellular Delivery of Lipophilic and Water-Soluble Payloads. *ACS Appl. Mater. Interfaces* **2018**, *10* (40), 33857–33866. https://doi.org/10.1021/acsami.8b09906.

(2) A. Napoli; N. Tirelli, *; G. Kilcher, and; Hubbell, J. A. *New Synthetic Methodologies for Amphiphilic Multiblock Copolymers of Ethylene Glycol and Propylene Sulfide*. ACS Publications. https://doi.org/10.1021/ma0108057.

(3) Bobbala, S.; Allen, S. D.; Scott, E. A. Flash Nanoprecipitation Permits Versatile Assembly and Loading of Polymeric Bicontinuous Cubic Nanospheres. *Nanoscale* **2018**, *10* (11), 5078–5088. https://doi.org/10.1039/C7NR06779H.

(4) Markwalter, C. E.; Prud'homme, R. K. Design of a Small-Scale Multi-Inlet Vortex Mixer for Scalable Nanoparticle Production and Application to the Encapsulation of Biologics by Inverse Flash NanoPrecipitation. *Journal of Pharmaceutical Sciences* **2018**, *107* (9), 2465–2471. https://doi.org/10.1016/j.xphs.2018.05.003.

(5) Hopkins, J. B. BioXTAS RAW 2: New Developments for a Free Open-Source Program for Small-Angle Scattering Data Reduction and Analysis. *J Appl Cryst* **2024**, *57* (1), 194–208. https://doi.org/10.1107/S1600576723011019.

(6) Zheng, S.; Wolff, G.; Greenan, G.; Chen, Z.; Faas, F. G. A.; Bárcena, M.; Koster, A. J.; Cheng, Y.; Agard, D. A. AreTomo: An Integrated Software Package for Automated Marker-Free, Motion-Corrected Cryo-Electron Tomographic Alignment and Reconstruction. *Journal of Structural Biology: X* **2022**, *6*, 100068. https://doi.org/10.1016/j.yjsbx.2022.100068.

(7) Kremer, J. R.; Mastronarde, D. N.; McIntosh, J. R. Computer Visualization of Three-Dimensional Image Data Using IMOD. *Journal of Structural Biology* **1996**, *116* (1), 71–76. https://doi.org/10.1006/jsbi.1996.0013.

(8) Dragonfly 2024.1 [Computer Software]. Comet Technologies Canada Inc., Montreal, Canada; Software Available at Https://Dragonfly.Comet.Tech/.
